# Herpes Zoster Vaccination: Insights into Efficacy, Safety, and Guidelines

**DOI:** 10.3390/vaccines13050477

**Published:** 2025-04-28

**Authors:** Michał Oleszko, Paweł Zapolnik, Hanna Czajka

**Affiliations:** College of Medical Sciences, University of Rzeszów, 35-315 Rzeszów, Poland; pawel.zapolnik@onet.pl (P.Z.); hanna.czajka@onet.pl (H.C.)

**Keywords:** herpes zoster, chickenpox, communicable disease, infections, immunology, vaccines

## Abstract

Background: The varicella–zoster virus (VZV) is a human herpesvirus that primarily causes varicella (chickenpox) as an initial infection, characterized by distinctive skin lesions. It can later reactivate, leading to herpes zoster (shingles). Once reactivated, VZV infection may result in serious complications, the most common being postherpetic neuralgia. Fortunately, vaccination can prevent this condition. Objectives: In this study, we provide a comprehensive analysis of zoster vaccines, including clinical trials, safety profiles, and reimbursement guidelines across various countries. Results: Our findings confirm the vaccine’s effectiveness and safety across diverse populations, aligning with previous clinical trials and real-world data, and summarize global vaccination guidelines.

## 1. Zoster Vaccine Live

Herpes zoster (HZ) is a vaccine-preventable disease. Two vaccines, the recombinant zoster vaccine (RZV; brand name: Shingrix) and the zoster vaccine live (ZVL; brand name: Zostavax), have been developed and introduced for public use. The first vaccine introduced was a live attenuated vaccine that was licensed in 2006 for the prevention of herpes zoster [1,2].

ZVL vaccination leads to an increase in varicella–zoster virus (VZV)-specific CD4+ T cells, peaking between 1 and 4 weeks after immunization [3]. These CD4+ T cells demonstrate broad reactivity to several functionally significant VZV proteins, including structural glycoproteins such as gE, gB, gH, gM, and gI. In summary, these findings suggest that immunization with ZVL results in an increase in the population of polyfunctional memory CD4+ T cells [4,5,6,7].

Initially, it was approved for use in adults aged 60 and older [1], with the indication later expanded to include adults aged 50 and older in 2011 [8]. However, the US Advisory Committee on Immunization Practices (ACIP), which recommended the routine use of the live zoster vaccine for those aged 60 and above in October 2006, has never extended this recommendation to the 50–59 age group, partly due to concerns about the duration of protection [9]. Two key phase III efficacy studies, the Shingles Prevention Study (SPS) [10] and the ZOSTAVAX Efficacy and Safety Trial (ZEST) [11], showed that ZVL was 51% effective against HZ in individuals aged 60 and older and 69.8% effective in those aged 50–59 [8]. The efficacy was lower in adults aged 70 and above (37.6%) [12] and decreased further to 18% in those aged 80 and older [13].

Due to the use of an attenuated live virus as the active ingredient, the vaccine is contraindicated in individuals undergoing immunosuppressive therapy or with primary and acquired immunodeficiency conditions, such as leukemia, lymphomas, immunosuppression due to HIV/AIDS, and cellular immune deficiencies, as well as in pregnant women and those with active tuberculosis [8]. The administration of ZVL may increase the risk of burden of HZ in those with a debilitated immune system [12], including cases of death after vaccination in immunocompromised (IC) patients [8,13], which greatly limits the applicability of the live vaccine. Serious adverse effects (AEs) occurred in elderly non-immunocompromised (non-IC) patients as well [14].

By 2017, when RZV was licensed [15], ZVL was available in over 55 countries, with more than 34 million doses distributed globally [16]. As of 2023, over 50 million people have received the ZVL vaccine [17].

## 2. Recombinant Zoster Vaccine

### 2.1. General Information

In 2017, the Food and Drug Administration (FDA) approved RZV, a recombinant vaccine without a live component, consisting of a variant of VZV’s glycoprotein E (gE) and the AS01B adjuvant system. In 2018, it was endorsed by the Centers for Disease Control and Prevention (CDC), and, in the same year, the ACIP updated its guidelines for the use of RZV in adults aged 50 and above [18,19,20]. In 2021, the FDA broadened indications for RZV to include IC individuals aged 18 or more, and, in the same year, the ACIP endorsed a two-dose regimen for IC patients aged 19 and above [21].

RZV is designed to induce anti-VZV immunity through gE, while the AS01B adjuvant system plays a critical role in both shaping and enhancing the immune response. gE is a crucial part of the development of the immune response, as it is abundantly present on VZV-infected human cells. Moreover, it is a target for immune cells, including T cells, and antibodies [22].

The AS01B adjuvant system stimulates immunogenicity because it comprises both the saponin QS21 derived from *Quillaja Saponaria*, an evergreen tree native to Chile, and the toll-like receptor type 4 (TLR4) agonist, 3-O-desacyl-4′-monophosphoryl lipid A (MPL). QS21 and MPL function synergistically to promote a significant increase in CD4+ cell levels and stimulate antibody responses [23]. RZV can increase the level of B cells [24,25], which have a crucial role in enhancing the immune system by producing antibodies.

RZV is given as a two-dose intramuscular vaccine, with the second dose administered 2 to 6 months after the first. It is also applicable to persons who have previously received ZVL if a minimum of 2 months has elapsed since that vaccination [26].

For IC individuals requiring quicker immunity, an accelerated schedule can be used, with two doses spaced at least one month apart. If more than six months have elapsed since the initial dose, the regimen does not need to be restarted; the second dose should be given as soon as possible. If it is administered before 4 weeks after the initial dose, the following dose must be given again [27].

The simultaneous administration of RZV with the standard adult dose does not markedly affect the tolerability, immune response, or overall safety of any of these vaccines, so they can be administered at once [28], including the coronavirus disease 2019 (COVID-19) mRNA booster vaccine [29]. Moreover, some recent studies indicate that RZV can delay or prevent dementia in elderly patients [30]. In the study by Eyting et al. [31], the risk of dementia was reduced by 3.5 percentage points over a follow-up period of 7 years (95% confidence interval (CI) = 0.6–7.1, *p* = 0.019), with a stronger effect in females compared to males. In the retrospective cohort study by Tang et al. [32] (n = 4,502,678 persons, including 206,297 partially vaccinated and 460,413 fully vaccinated), RZV significantly reduced the risk of dementia for two doses (hazard ratio (HR): 0.68; 95% CI: 0.67–0.70; *p* < 0.001) and for one dose (HR 0.89; 95% CI: 0.87–0.92; *p* < 0.001).

The differences between ZVL and RZV are listed in Table 1.

### 2.2. Phase Trials

Initially, RZV was approved for the prevention of HZ in adults aged ≥50, following phase III clinical trials (ZOE-50 and ZOE-70) conducted in individuals aged ≥50 years and ≥70 years, respectively [33]. It was licensed in 2017 in the USA [34,35] and Canada [36], and subsequently in 2018 in the European Union, Japan [37], and Australia [38].

The results from these studies revealed that the vaccine demonstrated substantial efficacy rates, reaching 97.2% (95% CI: 93.7–99.0) in individuals aged 50 years and older, with a mean follow-up period of 3.2 years, and 91.3% (95% CI: 86.8–94.5) in those aged 70 years and above, with a mean follow-up duration of 3.7 years. Commonly reported AEs included mild to moderate transient reactions both at the injection site and systemically [39,40].

In 2021, the indication for RZV was broadened to include persons aged ≥18 years who are at an elevated risk for HZ due to immunodeficiency or immunosuppression resulting from known medical conditions or treatments [41,42]. In that same year, ACIP revised its recommendations to endorse a two-dose regimen of RZV in adults aged 19 years and older who experience immunodeficiency or immunosuppression [27,43]. Within the European Union, the vaccine received licensing in 2018 for use in individuals aged 50 years and older. This approval was later extended in 2020 to include patients ≥18 years who are at an elevated risk of developing HZ [21,44].

In the trial ZEO-LFTU [45] conducted in 18 countries worldwide, regarding the final analysis of efficacy and safety after 11 years of follow-up (n = 7258, n = 2046 50–59 Y, n = 1243 60–69 Y, n = 3984 ≥ 70 Y), with a mean age of 67.3 (± 9.5), the general VE against the first or only episode of HZ was 79,77% and, in the group of patients within 50–59 Y, 60–69, and ≥70 Y, it was 86.67%, 81.74%, and 73.18%, respectively.

To compare, the overall vaccine efficacy from 1-month post-dose 2 in ZOE-50/70 studies for individuals ≥50 Y was 87.73% (95% CI: 84.89–90.12) and, in the group of patients within 50–59 Y, 60–69, and ≥70 Y, it was 91.74%, 92.57%, and 84.33%, respectively.

The safety profile remained clinically acceptable, and no new adverse reactions were identified in ZOE-LTFU. No deaths or serious AEs were considered to have a connection with vaccination.

China was not included in ZOE-50/70 trials; therefore, data assessing the Chinese population were missing [46]. In a phase IV randomized, placebo-controlled, observer-blind study, the population comprised a non-IC population ≥ 50 years. Over a mean follow-up of 15.2 (±1.1) months, 31 cases of HZ were confirmed (RZV group: 0; placebo group: 31), resulting in an IR of 0.0 and 8.2 per 1000 PY, respectively. The overall vaccine efficacy was 100% (CI: 89.82–100). For those aged 50–69, the vaccine efficacy was 100% (CI: 85.29–100), while, for participants aged 70 or older, it was 100% (CI: 60.90–100).

### 2.3. Licensure in Immunocompromised Patients

The efficacy of RZV in specific populations, particularly those with significant immunosuppression, including individuals who were previously ineligible for ZVL, is still being investigated. Unlike ZVL, which is contraindicated in patients with moderate to severe immunosuppression, RZV does not exclude those persons from receiving the vaccine [47,48].

The FDA based its licensure of RZV in IC patients on two trials [49]. The first of them was a randomized, placebo-controlled study in almost 1850 IC individuals ≥18 years old who have become auto-hematopoietic stem-cell transplantation (HSCT) recipients within 50–70 days before vaccination. The second one was a post hoc analysis of a study in almost 570 adults who were IC due to the hematologic therapy. Both studies proved that RZV effectively decreased the incidence rate (IR) of HZ occurring ≥1 month after the second dose of immunization [50,51], and AEs were similar to non-IC patients, including, e.g., injection site pain, redness, swelling, headache, shivering, fever, myalgia, and fatigue [52].

In the first of the referenced trials [50], over a median follow-up period of 21 months, at least one episode of HZ was confirmed in 49 individuals who were given the vaccine and 135 individuals who received a placebo, resulting in a vaccine efficacy of 68.2%. There were significant reductions in the incidence of postherpetic neuralgia (PHN), other predefined HZ-related complications, and the duration of severe pain associated with HZ. The efficacy observed in individuals who underwent transplantation was lower compared to those who were not after transplantation at age ≥50 years [35,36], possibly due to a debilitated immune response linked to hematologic conditions and the high-dose preparative regimens administered before (HSCT). However, the overall efficacy in those who were given at least one dose was comparable to that of a heat-inactivated VZV vaccine in a comparable population.

In the trial involving HSCT recipients [51], participants were randomly assigned to receive the RZV or a placebo. By month 2, 119 of 148 participants in the vaccine group exhibited a humoral response, compared to just 1 of 130 participants receiving placebo. The concentration of anti-glycoprotein E antibodies was significantly higher in the vaccine group across all patients, excluding those with non-Hodgkin B-cell lymphoma and chronic lymphocytic leukemia. The immune responses, both humoral and cell-mediated, remained above baseline levels in all groups until the 13th month. As expected, the reactogenicity was higher in the reactogenic RZV group than in the placebo group, particularly causing pain and fatigue. Despite this, the incidence of unsolicited or serious adverse events, possible immune-mediated diseases, disease-related adverse events, and fatal serious adverse events was comparable in both groups.

### 2.4. Effectiveness

#### 2.4.1. Effectiveness in Individuals Aged ≥50

Two real-world retrospective cohort studies from the US included only non-IC patients ≥ 50 years [35,53,54]. The first one by Sun et al. analyzed data from Hawaii, USA (n = 78,356 patients, of which 11,864 received two doses of RZV) [54], and the second one by Sun et al. analyzed data from Massachusetts, USA (n = 4,769,818 adults, of which 173,745 were vaccinated in a two-dose regimen) [53].

In the study from Massachusetts by Sun et al. [53], the HZ incidence rate was 258.8 cases per 100,000 person-years (PY) in vaccinated individuals (95% CI, 230.0–289.4) compared to 893.1 (95% CI, 886.2–900.0) in those unvaccinated. RZV showed an overall efficacy of 85.5% (95% CI, 83.5–87.3%), with 86.8% (95% CI, 84.6–88.7%) effectiveness in individuals aged 50–79 years and 80.3% (95% CI, 75.1–84.3%) in those aged 80 or older.

In the Hawaiian study by Sun et al. [54], the IR among the vaccinated group was higher and amounted to 325.6 (95% CI: 217.7 to 464.4) cases per 100,000 PY, compared to 1063.3 cases per 100,000 PY (95% CI: 1006.0 to 1122.8) among those unvaccinated. The vaccine effectiveness (VE) of 76.1% reported in this clinical trial was considerably lower than the results of two earlier clinical studies, where the vaccine efficacy was reported to be 97% in individuals 50 years and older [39] and 90% in persons ≥ 70 years old [52].

In the systematic review and meta-analysis by Zeevaert et al. [55], involving 23 articles regarding 14 studies (therein ZOE-50 and ZOE-70, both in a non-IC population and four randomized clinical trials on IC patients), the efficacy of RZV in preventing HZ and PHN was statistically significant among immunocompetent older individuals, with the VE reaching 94% and 91.2% in persons aged ≥50 years, and 91.3% and 88.8% in those aged 70 and older.

Meta-analyses conducted by McGirr et al. [56] and Tricco et al. [57] comparing both vaccines proved that RZV has a significantly higher effectiveness in preventing HZ [57] and PHN [56] than ZVL in 60-year-old adults or older [36]. The studies on the effectiveness of RZV in people aged ≥ 50 are listed in Table 2.

**Table 2:** Effectiveness of RZV in people aged ≥50.

Publication	Study Type	Sample Size	Results
Sun et al. [54]	Retrospective cohort study	78,356 patients Vaccinated: 11,864 Unvaccinated: 66,492	IR per 100,000 PY: Vaccinated: 325.6 Unvaccinated: 1063.3 VE: 76.1%
Sun et al. [53]	Retrospective cohort study	4,769,818 patients Vaccinated: 173,745 Unvaccinated: 4,596,074	IR per 100,000 PY: Vaccinated: 258.8 Unvaccinated: 893.1 VE: 85.5 %
Zeevaert et al. [53]	Systematic review and meta-analysis	-	VE: 94%
McGirr et al. [56]	Meta-analysis	-	VE: 92% (RZV vs. ZVL; people aged ≥60)
Tricco et al. [57]	Meta-analysis	-	Vaccine efficacy: 94% (RZV vs. placebo) 85% (RZV vs. ZVL)

Abbreviations: IR—incidence rate; PY—person-years; RZV—recombinant zoster vaccine; VE—vaccine effectiveness; ZVL—zoster vaccine live.

#### 2.4.2. Effectiveness in IC Patients and with Comorbidities

A recent study by Curran et al. [58] indicates that RZV vaccination is projected to prevent cases of HZ and its associated complications in patients with specific cancers, such as hematopoietic stem cell transplant recipients, individuals with breast cancer (used as an example of a solid tumor), and those with Hodgkin’s lymphoma, over a 30-year time horizon. The analysis was based on the assumption that HSCT recipients would remain immunocompromised for 5 years, drawing from epidemiological data on HZ in autologous HSCT patients [59].

A combined analysis by Silverii et al. [60] of studies assessing RZV against a placebo demonstrated a significantly lower risk of HZ incidence in patients with diabetes mellitus (95% CI 0.04–0.19). There were no observed differences in the rates of severe adverse events or mortality between the groups.

In another meta-analysis by Tue Xia et al. including 17 RCTs and 19 cohort studies [61], in IC individuals, RZV demonstrated superior efficacy compared to ZVL, showing a relative vaccine efficacy of 84% (95% CI: 53–95%) and a relative VE of 49% (95% CI: 21–67%). This advantage was consistent across genders and in subjects aged 60 years and older.

The systematic review and meta-analysis by Mbinta et al. [62] regarding the post-licensure VE of RZV was 64.1% in IC individuals (95% CI: 57.2–69.8).

Several randomized clinical trials and observational studies included in the systematic review and meta-analysis by Zeevaert et al. [55] showed VE in in IC patients. High-quality randomized controlled trials demonstrated an efficacy of 68.2% in individuals post-HSCT aged ≥18 years (95% CI: 55.6–77.5) [50], and a post hoc evaluation in patients with HM indicated an effectiveness of 80.4% (95% CI: 73.1–86.5) [45]. In two observational studies, the VE in immunocompromised individuals was comparable to the efficacy observed in the ZOE-HSCT trial and the randomized study involving patients with hematologic malignancies (unadjusted VE of 65% vs. 70%). However, the actual benefit observed in real-world settings was smaller—a reduction of 106 cases of HZ per 10,000 PY (58,59) compared to 618 fewer cases in the clinical trials.

Moreover, apart from being effective in IC individuals, RZV can reduce mortality in vulnerable patients. The study assessing the influence of RZV administration to individuals with immune-mediated inflammatory diseases (like RA, spondylarthritis, or psoriasis) treated with Janus kinase inhibitors [63] showed that RZV administration significantly reduced all-cause mortality (HR 0.610, 95% CI: 0.427–0.870), especially in females (HR 0.585, 95% CI: 0.379–0.901) and individuals aged ≥65 (HR 0.500, 95% CI: 0.301–0.806). The studies on the effectiveness of RZV in IC patients and patients with comorbidities are listed in Table 3.

**Table 3:** Effectiveness of RZV in IC patients and patients with comorbidities.

Publication	Study Type	Results
Silverii et al. [60]	Systematic review and meta-analysis	IR per 1000 PY in patients with diabetes: Vaccinated: 0.8 Placebo: 9.1
Xia et al. [61]	Systematic review and meta-analysis	VE: 63% (RZV vs. unvaccinated) Relative VE: 45% (RZV vs. ZVL)
Mbinta et al. [62]	Systematic review and meta-analysis	VE: 64.1%
Zeevaert et al. [55]	Systematic review and meta-analysis	VE in HSCT recipients: 68.2% VE in HM: 80.4% (RCT studies) VE in HM: 65% (observational studies)

Abbreviations: IC—immunocompromised; IR—incidence rate; HM—haematological malignancies; HSCT—hematopoietic stem cell transplantation; PY—person-years; RZV—recombinant zoster vaccine; VE—vaccine effectiveness; ZVL—zoster vaccine live.

#### 2.4.3. Effectiveness Depending on the Dose Regimen

The real-world observational study by Izurieta et al. [54] confirmed the effectiveness of the two-dose regimen, although the effectiveness was lower than in the clinical trials, and amounted to 70.1% (95% CI, 68.6–71.5) for those who received two doses and 56.9% (95% CI, 55.0–58.8) for individuals who received only one dose.

The effectiveness of RZV against HZ depending on the dosage regimen was a subject of the prospective cohort study from 2024 by Zerbo et al. [64], encompassing approximately 2 million individuals who collectively provided 7.6 million PY of follow-up data. For individuals receiving one dose, the VE was 64%, while, for those who completed the two-dose regimen, the VE was 76%. Among participants who had taken corticosteroids before vaccination, the VE was 65%, compared to 77% in those who had not. While the two-dose effectiveness remained consistently high over the four-year follow-up, it was slightly lower than earlier trial results. However, the VE after one dose dropped significantly after the first year, emphasizing the critical importance of administering the second dose. The studies on the effectiveness of RZV depending on the dose regimen are listed in Table 4.

**Table 4:** Effectiveness of RZV depending on the dose regimen.

Publication	Study Type	1 Dose	2 Doses
Izurieta et al. [54]	Real-world observational study	VE: 56.9%	VE: 70.1%
Zerbo et al. [64]	Prospective cohort study	VE: 64%	VE: 76%

Abbreviations: IR—incidence rate; PY—person-year; RZV—recombinant zoster vaccine; VE—vaccine effectiveness.

#### 2.4.4. Influence of Sex on Post-RZV Immune Response

Some studies assess the immunogenicity and effectiveness of RZV regarding sex. The analysis by Willer et al. [65] showed a similar efficacy of RZV in both males and females. In the pooled ZOE-50/70 population aged ≥70, the efficacy of the two-dose regimen of RZV against PHN amounted to 91.5% in men (95% confidence interval [CI]: 65.7–99.1), while, in women, it was 83.3% (95% CI: 24.8–98.2) (23). According to Losa et al. [66], sex does not significantly influence immune response rates. The post-RZV antibody avidity was not affected by different sexes in both the study by Weinberg et al. [67] and in the study by Schmid et al. [68]. However, the study by Lindemann et al. [69] showed that the male sex significantly induces VZV-specific cellular immunity in kidney transplant recipients, and, in the study by Koldehoff et al. [70], the male sex was significantly associated (*p* ≤ 0.02) with an enhanced VZV-specific immunity in adult allogenic HSCT recipients.

### 2.5. Safety

RZV, being a non-live vaccine without preservatives, has very few absolute contraindications. The primary ones include a history of anaphylaxis following a previous dose of the vaccine and the presence of an acute illness at the time of administration [18]. However, the meta-analyses show an increased risk of adverse effects following immunization (AEFIs) compared to ZVL, but they are usually mild and local [56,57].

In prelicensure trials, 85% of the 6773 participants who received RZV reported experiencing local or systemic reactions, with around 17% reporting grade 3 reactions (defined as erythema or induration above 3.5 inches, or systemic symptoms that affect daily activities) [71]. Despite this, the occurrence of serious AEFIs, such as hospitalizations, life-threatening conditions, death, or influence on the fetus, was comparable between the RZV and placebo groups [71].

There are several large analyses assessing the safety profile of RZV (a few with a sample above one million RZV doses—both retrospective [72] and prospective [72]). Numerous other studies evaluated the safety of RZV [46,73,74]. Many of them included IC individuals [21,44,66,67,68,69,70,71] and those suffering from immune-mediated diseases [73,75,76,77,78,79,80].

The analysis by Hesse et al. [71] evaluated records available in the Vaccine Adverse Event Reporting System (VAERS), a national US surveillance system for reporting adverse effect reports from patients, health care providers, and manufacturers [81]. The data were collected for the period from October 2017 to June 2018. VAERS received 4381 reports (136 reports per 100,000 doses), including 130 (3.0%) serious adverse events (4 serious AE per 100,000 doses). The most frequent side effect (1034; 23.6%) was pyrexia. Among other common side effects, there were mild and transient systemic symptoms, like, e.g., fatigue, chills, and myalgia, as well as local injection site reactions. Seven fatal cases were described (range of age: 61–86 years), and the time from administration of the vaccine to death was in the range of 6 h to 6 weeks. Cardiovascular disease was the cause of death in four individuals, three of whom had multiple risk factors related to heart conditions. Septic shock resulted in the deaths of two immunosuppressed patients. No unexpected patterns were noticed among the AE reports, and the results were consistent with those observed in clinical trials.

The review by Tavares-Da-Silva et al. [20] assessing data from 15.638 AE reports submitted directly to GSK, the manufacturer of the vaccine, showed that the safety profile is aligned with the data obtained from clinical trials as well. After distributing an estimated 9.3 million doses by February 2019, most reports (95.3%) were categorized as non-serious. Reported cases mainly involved individuals aged 50–69 years (62.1%) and females (66.7%). Among the most common side effects, there were injection site reactions, as well as systemic reactions, including, e.g., pyrexia, fatigue, and chills.

The safety database was retrospectively analyzed for the period from October 2017 to April 2020 by Pirrotta et al. [82] in terms of cutaneous eruptions (e.g., the burden of HZ) following RZV administration. From 32,597,779 doses given worldwide, 2423 reports of HZ and its complications were reported, including 645 cases of possible vaccination failure (2.0 cases per 100,000 RZV doses). Reporting rates of HZ (n = 2344) were 7.19 per 100,000 doses, PHN (n = 92) 0.28 per 100,000 doses; herpes zoster ophthalmicus (HZO) (n = 81) 0.25 per 100,000 doses, and HZ oticus (n = 12) 0.04 per 100,000 doses. There were 1928 cases of possible virus reactivation. However, the statistical analysis of these reports revealed that the number of observed cases was lower than would be expected in the general population without vaccination. Moreover, 810 cases of non-HZ vesicular and bullous skin lesions, including injection site reactions, were observed.

In another retrospective study with a sample of above 1,000,000 doses (January 2018–May 2020; 1,014,329 RSV doses) [83], the findings corresponded to the clinical trials. There was no evidence suggesting a correlation between RZV and serious AEs.

In the prospective study from the UK by Nelson et al. [72] (647,833 doses given from January 2018 to December 2019), data regarding AEs after RZV administration were studied to identify the risk of ten serious medical conditions following vaccination, including Guillain–Barré syndrome, stroke, acute myocardial infarction, and anaphylaxis. No increased risk in any of the studied outcomes was detected.

Baumrin et al. [84] conducted an observational prospective cohort study where they evaluated AEFIs in individuals after allogenic hematopoietic cell transplantation, and the vaccine was found to be safe and well tolerated. From December 2018 to June 2020, a total of 158 individuals (mean age of 55 years) received at least one dose (total vaccinated cohort), and 150 participants (95%) completed the two-dose series (modified total vaccinated cohort). The majority (92.1%) experienced AEs, primarily pain at the injection site, reported by 86% of participants. The IR of graft-versus-host-disease was comparable to other studies (adjusted IR ratio of 1.05, 95% CI: 0.8–1.38). Four individuals (2.5%) in the total vaccinated cohort and three (28.3/1000 PY) in the modified cohort developed HZ.

The safety profile of RZV in individuals with immune-mediated diseases was the subject of the study by Leung et al. [76] The study covered the database of the IBM MarketScan (55.645 individuals between 50 and 64 years) and Medicaid Service Medicare (160,545 patients ≥ 65 years). There was no evidence of a higher rate of flares in the studied cohort following RZV vaccination after the first and second doses.

Moreover, Bruxvoort et al. assessed data from Kaiser Permanente Southern California for the association between RZV and COVID-19 outcomes and found that RZV may have a protective influence on the burden of COVID-19 infection [85].

Some concerns regarding RZV influence on flares in patients with immune-mediated diseases, including inflammatory bowel diseases, have been raised [78]. In the prospective observational study evaluating the safety of RZV in patients with inflammatory bowel disease in the USA [78], which included 67 patients, rates of AEFI corresponded to the results from ZOE-50/ZOE-70. Only one case (1.5%) of a flare of inflammatory bowel disease was seen. The self-controlled case series analysis of the risk of flares among people with immune-mediated inflammatory diseases in the US [76] did not show a greater rate of flares in individuals ≥50 years after RZV. Also, in a retrospective study assessing safety with the same diseases as above, RZV vaccination was not connected with a higher probability of a flare [80].

In the Italian study by Venerito et al. [73], local reactions were the most frequently reported AEFIs, occurring in 37.44% of completed follow-ups (386/1031). The most common cause of vaccination was an ongoing immunosuppressive therapy (54.65%). Systemic reactions were noted in 13.97% (144/1031), fever in 10.09% (104/1031), and neurological symptoms in 4.75% (49/1031) of diaries. There were four serious AEFIs reported, occurring at a rate of 0.38 per 100 participants. Factors such as older age, male sex, and a history of cardiovascular diseases were associated with a reduced risk of AEFIs (OR: 0.71; 95% CI: 0.52–0.98; *p* < 0.05), whereas having endocrine–metabolic disorders was linked to a higher risk (OR: 1.61; 95% CI: 1.15–2.26; *p* < 0.05).

In the Chinese study by Xia et al. [61] including 6138 patients, solicited adverse events occurred more frequently in the RZV group compared to the placebo group, with a median duration of 1–3 days for both. Pain at the injection site and fatigue were the most frequent AEs reported (72.1% and 43.4% for RZV; 9.2% and 5.3% for the placebo, respectively). No significant differences were noticed between the placebo and RZV groups in the case of unsolicited AEs, serious AEs, immune-mediated diseases, and deaths.

The study conducted in Italy by Constantino et al. [86] with 271 subjects (the majority were solid organ transplant recipients) showed that age, sex, and prior HZ significantly influenced the probability of AEFIs following immunization among frail individuals (e.g., with the presence of a severe condition that causes immunodeficiency, geriatric frailty, or comorbidities, like diabetes or autoimmune diseases). Females were more likely to experience mild and moderate AEFIs after the first dose (such as redness or swelling at the injection site and fatigue) (*p* = 0.02), but with no differences in duration. Moreover, in young individuals aged 28–39 and patients aged 40–65, systemic AEFIs occurred more frequently than in the elderly population aged ≥65, probably due to immunosenescence in older individuals. Elderly patients had a higher probability of local AEFIs, such as redness or swelling (*p* = 0.01). No differences in the duration of AEFIs were observed between these groups. A prior episode of HZ was also significantly associated with the highest risk of AEFIs following the second dose of RZV (*p* = 0.001). In this observational real-life study, 37% of patients suffered from AEFIs, most commonly pain at the injection site, both after first and second doses. The most serious AEFI was a systemic skin rash in one person.

Another study, conducted by Stefanizzi et al. [74], confirms that a male sex and older age reduce the risk of AEFIs. The study included 538 patients, mostly onco-hematological patients and patients suffering from cardiovascular diseases, who received a total of 1031 doses of RZV, of which 441 AEFIs were reported (42.7%), most commonly injection site local effects, malaise, asthenia, and fever. Males were less likely to have AEFIs (odds ratio vs. female sex: 0.72; 95% CI: 0.54–0.95; *p* < 0.05), as well as younger patients compared to the elderly population (age was inversely associated with AEFIs; OR: 0.97; 95% CI: 0.96–0.98; *p*-value < 0.001). Similar results regarding sex were obtained in the study by Costantino et al. (30), in which females reported AEFIs more often than males after both first (47% vs. 32%; *p* = 0.01) and second doses of RZV (51% vs. 36%; *p* = 0.03).

The post-marketing safety surveillance for RZV, analyzing AE reports of RZV in the US [87], showed that, from October 2017 to April 2024, 1,279,596 AEs were received by VAERS, including 97.3% classified as non-serious. However, 145 cases of GBS were reported, including two deaths. Overall, 86 reports of death following RZV administration were identified. Most fatalities were connected to underlying conditions, and there is no substantial evidence of RZV involvement in these deaths.

Despite RZV being safe, also in fragile patients, the FDA issued a caution regarding a heightened risk of Guillain–Barré syndrome following vaccination [88] as some post-marketing data indicate a possible elevated risk of the disease after vaccination; however, the existing data are still insufficient [72,89] and the relatively minimal risk should be evaluated against the significant health advantages, especially in IC patients [35,89]. However, patients and physicians should know the risk of GBS; therefore, in March 2021, the FDA highlighted the issue, putting the black box warning on RZV with information on the possible risk of GBS. The studies assessing AEFIs among RZV recipients are listed in Table 5.

**Table 5:** Studies assessing adverse effects among RZV recipients.

Publication	Study Type	Sample Size	Main Results
Hesse et al. [71]	Post-licensure safety surveillance	Approximately 3.2 million doses	Number of reported AEs: 4381 Serious AEs: 130 (3% of AEs)
Tavares-Da-Silva et al. [20]	Post-marketing safety surveillance review	9,323,118 doses	Number of reported AEs: 15,636 Serious AEs: 741 (4.7% of AEs)
Yih et al. [83]	Retrospective data-mining study	1,014,329 doses	No significant correlation between RZV and serious AEs
Baumrin et al. [84]	Prospective observational cohort study	158 HSCT recipients (150 patients received two doses; 8 patients received one dose)	No significant correlation between RZV and serious AEs, including graft-versus-host disease
Leung et al. [76]	Retrospective cohort study	216,199 patients	No significant correlation between RZV and flares in immune-mediated diseases
Bruxvoor et al. [85]	Retrospective cohort study	447,732 patients	RZV may have a protective influence on the risk of burden of COVID-19 infection and the risk of hospitalization due to COVID-19 (16% and 32% lower risk, respectively)
Satyam et al. [78]	Prospective observational study	67 patients with IBD	No significant correlation between RZV and flares in IBD
Khan et al. [80]	Retrospective cohort study	1677 patients with IBD	No significant correlation between RZV and flares in IBD
Xia et al. [61]	Systematic review and meta-analysis	6138 patients	No significant correlation between RZV and serious AE
Constantino et al. [86]	Observational real-life study	271 frail patients (including 209 kidney transplant recipients)	No significant correlation between RZV and serious AEs; females were more likely to report mild and moderate AEs; systemic AEFIs were more likely in younger individuals compared with elderly ones
Stefanizzi et al. [74]	Retrospective population-based study	538 individuals, mostly onco-hematological patients and patients suffering from cardiovascular diseases	Male sex and older age reduce the risk of AEFIs
Shy et al. [87]	Post-marketing safety surveillance	1,279,596 AEs reported to VAERS	The safety profile of RZV is consistent with the data collected in clinical trials. There is a potential association between GBS and RZV

Abbreviations: AE—adverse effect; AEFIs—adverse effect following immunization; GBS—Guillain–Barre syndrome; IBD—inflammatory bowel disease; IR—incidence rate; HSCT—hematopoietic stem cell transplantation; PY—person-years; RA—rheumatoid arthritis; RZV—recombinant zoster vaccine; VAERS—Vaccine Adverse Event Reporting System; VE—vaccine effectiveness; ZVL—zoster vaccine live.

## 3. Guidelines and Reimbursement

Due to the superior efficacy of RZV over ZVL in preventing HZ infections and associated complications like PHN, numerous countries have changed ZVL to RZV within their national immunization programs and guidelines [38,53,90,91]. This transition has been implemented in countries such as the United States as of November 2020 and Australia as of November 2023 [34].

RZV was initially recommended for immunocompetent adults aged 50 and above. However, on July 23, 2021, the FDA expanded its indication, and, on 20 October 2021, the ACIP advised two doses of RZV for adults aged 19 and older with immunodeficiency or immunosuppression to prevent HZ and related complications [29]. The American Society of Clinical Oncology (ASCO) claims that RZV may be given in IC patients between 12 and 18 months after allo-HSCT and between 3 and 12 months after auto-HSCT [92].

In Australia, individuals aged 65 years and older and First Nations (Aboriginal and Torres Strait Islander) individuals aged ≥50 years, as well as immunocompromised patients aged ≥18 years, can receive a two-dose regimen of RZV at no cost through the National Immunization Program [34].

RZV is recommended in other well-developed countries, like in the UK [93] and in New Zealand, where ZVL is no longer imported [94].

The UK Joint Committee on Vaccination and Immunisation (JCVI) advised that the national HZ immunization program will be introduced in stages. Initially, the focus was on vaccinating individuals between ages 65 and 70 for five years. This would then expand to vaccinating those between 60 and 65 for another five years, with the goal of eventually offering routine vaccination to those reaching 60. Additionally, people aged 70 to 79 who have not yet been vaccinated, as well as those over 50 with severely weakened immune systems, are eligible for RZV until they turn 80 [95].

The National Vaccine Prevention Plan of Italy for the years 2022–2023 introduces a new recombinant zoster vaccine targeting individuals aged 65 and older, as well as at-risk individuals aged 50 and above who exhibit frailty or have chronic health conditions [96].

In Poland [97], vaccination against HZ is recommended for individuals aged over 50, those aged 18 and older receiving immunosuppressive therapy, and patients aged 18 and older with comorbidities that increase the risk of shingles (such as chronic heart, liver, lung, or kidney diseases, autoimmune disorders, diabetes, and depression). This especially includes those at higher risk who have regular and close contact with young children [97]. A separate expert opinion has been presented regarding solid tumors, encouraging those patients to get vaccinated [98]. It is recommended to give the first dose 2–3 weeks before systemic treatment to prevent the risk connected with leukopenia after chemotherapy, and to give the second dose 2 months after the first dose.

RZV is both recommended and provided at no cost to older adults or immunocompromised individuals in countries such as the United Kingdom [99], Italy [100], Luxembourg [101], and some regions of Spain [102,103,104]. Greece [105,106,107] subsidizes the cost of ZVL in all persons aged between 50 and 65, while RZV is free of charge for IC individuals aged ≥18.

In Germany, RZV was initially introduced in late 2018 [108]. Since March 2019, it has been covered for all insured individuals aged 60 and older, as well as for immunosuppressed individuals or those with certain comorbidities aged 50 and above [109].

In Belgium [110], as of 1 November 2023, RZV is reimbursed for individuals aged 18 and older in specific circumstances. These include those with hematological malignancy, a malignant tumor actively treated within the last five years, HIV infection, and those who have undergone organ or hematopoietic stem cell transplantation or are candidates for such transplants. In France [111], although most vaccinations are covered in 65% [112], the vaccination against HZ is covered only in 30% [113].

In Poland, the Agency for Health Technology Assessment and Tariff System decided to reimburse RZV at 50% [114], starting from 1 January 2024 for adults who belong to risk groups, including, among others, those with diabetes, chronic heart and lung diseases, chronic kidney failure, rheumatic diseases, and immunosuppression, and the reimbursement was expanded to 100% from 1 April 2025 [115] for patients aged 65 and older with the above comorbidities. In contrast, RZV is not reimbursed in countries like Austria and the Czech Republic [103].

In New Zealand [94], RZV is recommended for all persons aged 50 or older; however, it is reimbursed only during the 12 months after their 65th birthday, as well as for some IC patients.

In the United States, the Affordable Care Act (ACA) mandates that health insurance marketplace plans and most private insurance providers fully cover ACIP-recommended vaccines, including RZV. This coverage must be provided without any cost-sharing measures, such as deductibles, copayments, or coinsurance, if the vaccine is administered in-network. As a result, 96% of privately insured individuals can receive the RZV vaccine at no additional charge [116]. Countries that subsidize for RZV are listed in Table 6.

**Table 6:** Level of reimbursement in countries that offer subsidized RZV vaccines.

Country	Reimbursement Level	Group Covered by Reimbursement
Australia [34]	100%	Persons aged ≥65, First Nations people aged ≥50, persons ≥ aged 18 at higher risk of HZ
Belgium [110]	100%	Some groups of IC persons aged ≥18
France [111]	35%	Persons aged ≥65, IC persons aged ≥ 18
Germany [108]	100%	Persons aged ≥60, persons aged ≥50 at higher risk of HZ
Greece [105,106,107]	100%	Persons aged 60–75 years *; IC persons aged ≥18 **
Italy [96]	100%	Persons aged ≥65
Luxembourg [101]	100%	Persons aged ≥65 and IC persons aged ≥18
New Zealand [94]	100%	Persons at age 65 and some IC persons aged ≥18
Poland [115]	50–100%	50%: persons aged 18–64 at higher risk of HZ burden 100%: persons aged ≥65 at higher risk of HZ burden
Spain [102,103,104]	100% ***	Differentiated depending on the region
United Kingdom [95]	100%	Ultimately all persons aged ≥ 60 and IC patients
United States [116]	100% ****	All persons aged ≥50 and IC persons aged ≥19

* ZVL reimbursement; ** RZV reimbursement; *** some regions of Spain; **** in United States, the Affordable Care Act (ACA) requires health insurance marketplace plans and most private insurance providers to provide full coverage of ACIP-recommended vaccines, including RZV. Abbreviations: ACA—Affordable Care Act; ACIP—Advisory Committee on Immunization Practices; HZ—herpes zoster; IC—immunocompromised; RZV—recombinant zoster vaccine; ZVL—zoster vaccine live.

## 4. Conclusions

Our paper summarizes data regarding HZ occurrence, recurrence, and complications, and underlines the significance of RZV vaccination, especially among IC individuals and elderly patients at higher risk of HZ and its complications. The burden of HZ is more probable in these groups of patients, but vaccination may effectively prevent HZ. Both trials [39,40] and real-world [53,55] studies show an acceptable effectiveness and safety profile of RZV in persons aged ≥50 and patients with comorbidities or in a state of immunosuppression or immunodeficiency [49,50]. Many countries worldwide recommend and subsidize for RZV in elderly or IC patients [95,108,116]; however, in some countries, such as Greece [105,106,107], ZVL is still officially recommended and reimbursed. A review of the current knowledge of HZ and zoster vaccines in the Subcarpathian Province in Poland is the subject of our research and will be presented in the following papers. Despite strong evidence of effectiveness and a good safety profile of RZV, there is still space to increase awareness in patients about the importance of vaccination against HZ [117]. A review of the attitude toward vaccination against HZ among patients, as well as the knowledge of individuals susceptible to complications of HZ, is the subject of our research, and will be published in the near future.

## Figures and Tables

**Table 1:** Major differences between ZVL and RZV.

Vaccine	ZVL	RZV
Live/recombinant	Live	Recombinant
Main components	Lyophilized preparation of live, attenuated VZV	VZV’s gE and the AS01B adjuvant system
Recommendations	Persons at age ≥50 years	Persons at age ≥50 years and immunocompromised/immunosuppressed patients aged ≥18
Major contraindications	Immunosuppression, immunodeficiency, anaphylaxis to a vaccine ingredient; presence of an acute illness at the time of administration	Anaphylaxis following a previous dose of RZV or after a contact to any ingredient of the vaccine; presence of an acute illness at the time of administration
Year of licensure	2006	2017
Dosage	One dose (0.65 mL) intramuscularly or subcutaneously	Two doses (2 × 0.5 mL) intramuscularly, with 2–6 months between each dose

Abbreviations: gE—glycoprotein E, RZV—recombinant zoster vaccine; VZV—varicella–zoster vaccine; ZVL—zoster vaccine live.

## Data Availability

Not applicable.

## Abbreviations

The following abbreviations are used in this manuscript:

ACA	Affordable Care Act
ACIP	Advisory Committee on Immunization Practices
AE	Adverse effect
AEFI	Adverse effect following immunization
ASCO	American Society of Clinical Oncology
CDC	Centers for Disease Control and Prevention
CI	Confidence interval
COVID-19	Coronavirus disease 2019
FDA	Food and Drug Administration
HSCT	Hematopoietic stem-cell transplantation
HZ	Herpes zoster
HZO	Herpes zoster ophthalmicus
IC	Immunocompromised
JCVI	Joint Committee on Vaccination and Immunisation
IR	Incidence rate
MPL	3-O-desacyl-4′-monophosphoryl lipid A
Non-IC	Non-immunocompromised
PHN	Postherpetic neuralgia
PY	Person-years
RZV	Recombinant zoster vaccine
TLR4	Toll-like receptor type 4
VAERS	Vaccine Adverse Event Reporting System
VE	Vaccine effectiveness
VZV	Varicella–zoster virus
ZVL	Zoster vaccine live
